# Frequency–Geometry-Guided Network for Depth Map Super-Resolution

**DOI:** 10.3390/s26134282

**Published:** 2026-07-05

**Authors:** Zhiqiang Feng, Chong Zhang

**Affiliations:** Department of Automation, School of Computer Science and Artificial Intelligence, Beijing Technology and Business University, Beijing 100048, China; fzhiqiang99@126.com

**Keywords:** depth map super-resolution, geometric prior, RGB-guided depth restoration, spatial frequency domain, frequency-domain supervision

## Abstract

Depth super-resolution reconstructs high-resolution (HR) depth maps from low-resolution (LR) inputs with the aid of HR RGB guidance, but RGB edges often do not coincide with true depth discontinuities, causing texture copying and degraded geometric consistency. To address this problem, we propose Frequency–Geometry-Guided Network (FGGNet), a spatial–frequency fusion framework for RGB-guided depth map super-resolution. FGGNet introduces Multi-branch RGB-guided Convolution (MRGConv) to enhance RGB structural representations, a Geometry Prior-guided Fusion Module (GPFM) to filter geometrically inconsistent RGB responses using depth-derived priors, and radial complex spectral loss (RCSL) to emphasize boundary-related high-frequency components in the complex spectral domain. Experiments on NYU v2, Middlebury, Lu, and RGB-D-D show that FGGNet achieves competitive or superior reconstruction accuracy under synthetic and real-world degradation settings. Under the ×16 setting, FGGNet reduces RMSE by 13.7%, 22.8%, 18.5%, and 11.4% on the four datasets, respectively, compared with the average RMSE of five representative state-of-the-art methods. These results validate the effectiveness of combining geometric prior filtering with frequency-domain supervision for reliable depth reconstruction.

## 1. Introduction

High-quality depth maps are essential for applications such as 3D reconstruction, virtual reality, augmented reality, medical imaging, and industrial measurement [1]. Unlike color images, depth maps encode the geometric relationships within a scene. Their spatial resolution and structural fidelity therefore directly affect point-cloud reconstruction, boundary localization, and subsequent geometric inference. However, consumer-grade depth sensors are constrained by imaging mechanisms, sampling density, hardware cost, and power consumption. As a result, captured depth maps often suffer from low spatial resolution, blurred edges, local noise, and structural discontinuities. If fine structures and true boundaries in LR depth maps cannot be effectively restored, downstream systems may suffer from geometric distortion and measurement errors, which can further degrade perception reliability and decision-making robustness in complex scenes [2].

Guided depth map super-resolution (GDSR) has become a widely adopted paradigm for depth map super-resolution. It reconstructs high-resolution (HR) depth maps from low-resolution (LR) depth inputs with the aid of spatially aligned HR RGB images [1]. This paradigm is motivated by the rich texture, contour, and local contrast cues in RGB images, which can support depth-boundary recovery. Recent deep learning methods improve RGB–depth interaction from different perspectives. Coupled U-Net [3] uses dual-branch encoding and cross-layer interactions to model RGB–depth feature collaboration; AHMF [4] selects useful guidance through hierarchical multimodal attention; CTKT [5] transfers RGB-related scene structure knowledge from auxiliary tasks to depth super-resolution; and JIIF [6] formulates guided depth reconstruction as a joint implicit image function in continuous coordinate space. These studies show that deep learning has substantially improved RGB-guided depth reconstruction and has shifted the field from local filtering and hand-crafted priors toward end-to-end cross-modal representation learning.

However, RGB guidance is not always geometrically reliable. Prominent RGB edges do not always correspond to true depth discontinuities, particularly in texture-rich but geometrically flat regions. Directly transferring high-frequency RGB information may introduce color textures into the depth domain, leading to texture copying, boundary displacement, and artificial surface patterns. DCTNet [7] addresses cross-modal feature extraction and RGB texture over-transfer by combining discrete cosine-domain modeling, shared–private feature decomposition, and edge attention. From the perspective of cross-modal gaps, SUFT [8] further shows that not all high-frequency RGB information benefits depth recovery. It therefore uses an uncertainty-aware mechanism to suppress RGB features that are detrimental to HR depth reconstruction. These observations shift the central question from whether RGB guidance should be used to how geometrically valid RGB cues can be identified and unreliable texture responses suppressed before fusion.

Recent studies have attempted to address unreliable RGB guidance from the perspectives of progressive reconstruction, multi-scale structural modeling, self-supervised learning, and continuous representation. PDR-Net [9] reduces the difficulty of large-factor upsampling through progressive depth reconstruction. MIG-Net [10] strengthens boundary recovery by alternately exploiting intensity and gradient features. MMSR [11] models local cross-modal correlations through intermodulation under a self-supervised setting, while Wang et al. [12] exploit RGB–depth dependencies to reduce the reliance on clean paired supervision. DCSR [13] and CDF-DSR [14] further improve scale flexibility by introducing continuous depth representations and self-supervised RGB guidance. These methods broaden the task settings and reconstruction paradigms of GDSR. However, their main focus remains on reconstruction strategy, scale adaptation, or supervision design. The geometric reliability of RGB guidance itself is still not explicitly constrained, especially when high-frequency RGB textures are inconsistent with true depth boundaries.

A related line of research emphasizes structural selection and adaptation to real-world degradation. FDSR [2] establishes a real-world depth super-resolution benchmark on the RGB-D-D dataset and uses high-frequency components adaptively extracted from RGB images to guide depth restoration. RSAG [15] uses recurrent structural attention to select clear patterns and boundaries, thereby alleviating weak guidance caused by coarse feature concatenation. SFGNet [16] learns cross-modal structural flow between RGB and depth to recover structural distortions and suppress edge noise in real-world LR depth maps. DAD [17] combines anisotropic diffusion with deep neural networks to enhance edge propagation while preserving consistency with the input depth. SSDNet [18] decomposes shared and private features in a spherical feature space and uses contrastive learning to mitigate blurred edges, noisy surfaces, and RGB texture over-transfer. A2GSTran [19] employs asymmetric attention with guidance selection to enlarge the effective receptive field and improve the selectivity of RGB guidance. These methods mark a shift from indiscriminate fusion to selective structural transfer. However, most of them still rely on implicit feature space learning to estimate structural reliability, rather than using an explicit filtering mechanism derived from the current depth geometry.

Frequency-domain information provides a complementary perspective for depth structure recovery. Depth boundaries, surface details, and local geometric discontinuities are usually associated with high-frequency spectral components. Therefore, frequency-domain modeling can compensate for the limitations of spatial-domain convolution in recovering fine structures. DCTNet [7] reconstructs HR depth features in the discrete cosine domain, showing that frequency-domain representations can improve interpretability and reduce cross-modal texture mis-transfer in image-domain reconstruction. Qiao et al. [20] exploit explicit and implicit high-frequency features by combining local–global context modeling with frequency-domain projections for stage-wise HR depth recovery. SGNet [21] further shows that spatial-domain recovery alone is insufficient for compensating for missing high-frequency structures in LR depth maps. It therefore introduces both gradient-domain and frequency-domain modeling into depth map super-resolution, using the Gradient Calibration Module (GCM) and the Frequency Awareness Module (FAM) to enhance gradient structures and spectral high-frequency components, respectively. These studies indicate that high-frequency compensation is important for depth structure recovery. However, existing frequency-domain supervision often treats different spectral components with similar importance and does not sufficiently distinguish boundary-related high-frequency responses from general spectral errors.

**Figure 1:**
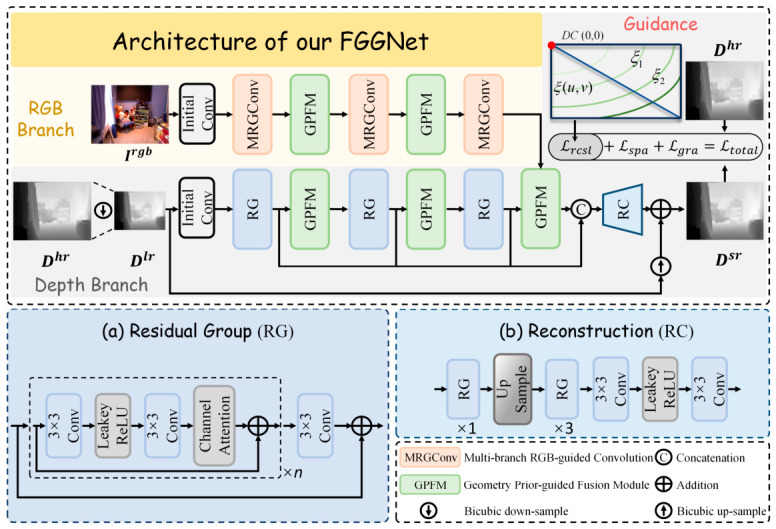
Overall architecture of FGGNet, including an RGB branch, a depth branch, multi-stage Geometry Prior-guided Fusion Module (GPFM) interactions, and a reconstruction module.

Based on the above analysis, we propose the Frequency–Geometry-Guided Network (FGGNet) for depth map super-resolution. The central idea of FGGNet is to treat RGB guidance as conditionally reliable structural information rather than as uniformly beneficial auxiliary input. SGNet [21] has shown that gradient and frequency cues are effective for recovering missing depth structures. However, its main objective is to enhance depth reconstruction by propagating RGB-derived gradient and frequency information into the depth branch. FGGNet addresses a different but complementary problem: before RGB structures are transferred, their consistency with the current depth geometry should be assessed. Therefore, FGGNet adopts the gradient prior from SGNet as structural support, and further introduces geometry prior filtering and radial complex spectral supervision to suppress texture over-transfer and improve boundary-oriented reconstruction. Figure 1 illustrates the overall architecture of FGGNet.

In summary, the main contributions are as follows:

- We propose a frequency–geometry-guided formulation for RGB-guided depth map super-resolution. Instead of treating RGB guidance as uniformly reliable, FGGNet explicitly distinguishes geometrically consistent structural cues from texture responses that may be harmful to depth reconstruction.
- We design a geometry-constrained RGB guidance mechanism. Multi-branch RGB-guided Convolution (MRGConv) first enhances RGB structural representations before fusion, while Geometry Prior-guided Fusion Module (GPFM) uses depth-derived geometric priors to gate RGB features before cross-modal interaction. This design changes RGB–depth fusion from direct feature injection to depth-geometry-constrained guidance selection.
- We introduce radial complex spectral loss (RCSL) for boundary-oriented frequency supervision. Different from amplitude–phase losses that treat spectral locations with similar importance, RCSL constrains the real and imaginary spectral components and assigns larger weights to high-frequency regions that are more closely related to depth discontinuities.
- Extensive experiments on NYU v2, Middlebury, Lu, and RGB-D-D demonstrate that FGGNet achieves competitive reconstruction accuracy under synthetic degradation and real-world degradation. The results further show that geometry prior filtering and frequency-domain supervision are complementary for suppressing RGB texture over-transfer and improving boundary fidelity.

The remainder of this paper is organized as follows: Section 2 reviews recent progress in guided depth map super-resolution and discusses related studies on RGB–depth fusion, real-world degradation modeling, and high-frequency structure reconstruction. Section 3 presents the proposed FGGNet, including the task formulation, overall network architecture, Multi-branch RGB-guided Convolution, Geometry Prior-guided Fusion Module, radial complex spectral loss, and other training losses. Section 4 reports the experimental settings, quantitative and qualitative comparisons, supplementary evaluation, statistical significance analysis, ablation studies, and complexity analysis. Section 5 discusses the relationship between FGGNet and SGNet, as well as the strengths of the proposed method. Finally, Section 6 concludes the paper and outlines the limitations of the proposed method and possible directions for future work.

## 2. Related Work

### 2.1. Guided Depth Map Super-Resolution

Zhong et al. [1] identify the selection of consistent structures from the guidance image and the suppression of inconsistent information as a central challenge in GDSR. Deep learning-based methods typically encode RGB and depth with dual- or multi-branch networks and then perform cross-modal fusion for structural transfer. Coupled U-Net [3] uses dual U-Net branches and cross-layer skip connections to enhance RGB–depth information exchange. Although this design is structurally simple and end-to-end trainable, its fusion process still relies mainly on feature concatenation, which limits its ability to suppress erroneous RGB textures. AHMF [4] propagates useful information across scales through multimodal attention and bidirectional hierarchical feature collaboration, reducing the gap between shallow details and deep semantics. However, its attention weights are still learned implicitly and lack explicit constraints derived from depth geometry.

Multitask learning and implicit representations have further expanded the paradigms of RGB-guided depth reconstruction. CTKT [5] uses depth estimation as an auxiliary task and distills scene structure knowledge from the RGB branch to the depth super-resolution branch through cross-task knowledge transfer, showing the value of structural priors for depth recovery. JIIF [6] formulates GDSR as a joint implicit image function, enabling finer-grained reconstruction in continuous coordinate space. MMSR [11] introduces mutual modulation for self-supervised cross-modal super-resolution, modeling cross-modal dependencies through source-to-guide and guide-to-source modulation. Wang et al. [12] exploit RGB–depth dependencies for self-supervised depth enhancement, improving depth quality without clean paired supervision. DCSR [13] performs continuous depth map super-resolution at arbitrary upsampling factors using local implicit guidance functions. CDF-DSR [14] further combines continuous depth-field modeling with self-supervised RGB guidance. Overall, these methods improve GDSR from the perspectives of cross-task transfer, implicit representation, self-supervised modulation, progressive reconstruction, and deformable or guided filtering. Their common limitation is that RGB–depth interaction is still mainly performed after feature extraction or during fusion. As a result, the structural quality and geometric reliability of RGB-guided features are not sufficiently regulated before they enter the depth reconstruction branch. This limitation motivates MRGConv, which strengthens RGB structural guidance at the feature-generation stage, and GPFM, which further filters RGB responses according to depth-derived geometric priors.

Progressive reconstruction and deformable modeling have been explored to address large-scale upsampling and cross-modal structural inconsistency. PDR-Net [9] progressively restores depth structures and uses adaptive feature recombination to fuse color and depth features, reducing the difficulty of large-scale reconstruction. SCD-Net [22] employs semi-coupled deformable convolution to model shared and modality-specific RGB–depth features, enabling more flexible cross-modal correspondence under local deformation. JGF [23] exploits deep hybrid-cross guided filtering to capture color–depth consistency and multi-scale structural cues for boundary recovery. Although these methods improve local alignment and feature interaction, cross-modal information is still mainly handled at the fusion stage, leaving the quality and geometric reliability of RGB-guided features insufficiently constrained. Our MRGConv addresses this limitation by improving RGB structural guidance at the feature-generation stage.

### 2.2. Structure Selection and Real-World Degradation Modeling

With the emergence of real-world benchmarks, GDSR research has shifted from reconstruction under ideal bicubic downsampling to structural distortion, edge noise, and cross-modal inconsistency in real-world LR depth maps. The RGB-D-D dataset [2] provides paired real-world LR and HR depth maps captured by mobile devices and high-precision sensors, revealing degradation patterns that differ substantially from synthetic downsampling. Therefore, assuming that RGB structures are always beneficial for real-world depth recovery can be unreliable. FDSR [2] establishes a real-world depth super-resolution baseline on this dataset and uses high-frequency components extracted from RGB images to guide depth recovery. Although FDSR advances real-world GDSR, it remains necessary to determine whether RGB high-frequency components are geometrically reliable for depth restoration.

Structure selection methods aim to mitigate excessive RGB texture transfer. SUFT [8] attributes harmful RGB texture transfer to cross-modality and resolution gaps, and uses a symmetric uncertainty-aware mechanism to filter unreliable guidance. RSAG [15] uses recurrent structure attention blocks to iteratively exploit the latest depth estimates and RGB features, selecting clear patterns and boundaries while alleviating weak guidance caused by coarse feature concatenation. A2GSTran [19] employs asymmetric attention with guidance selection to capture long-range dependencies and select effective guidance cues. Although these methods recognize the need for RGB guidance selection, most rely on attention learning in high-dimensional feature spaces, which limits interpretability and geometric specificity. In contrast, the proposed GPFM derives geometric edges from current depth features and combines them with positional priors to generate a gating map, explicitly linking RGB feature selection to depth geometry.

Real-world degradation modeling further highlights the importance of geometric consistency. SFGNet [16] observes that structural distortion and edge noise in real-world LR depth maps cause RGB–depth structural inconsistency, and learns cross-modality structure flow to guide RGB structural information transfer. DAD [17] combines anisotropic diffusion with deep neural networks, using diffusion-based edge propagation and network-based contextual reasoning to improve depth-boundary recovery. SSDNet [18] decomposes shared and private features in a spherical feature space and uses spherical contrast refinement to mitigate blurred edges, noisy surfaces, and excessive RGB texture transfer. These studies show that real-world GDSR must improve spatial resolution while preserving the geometric consistency of the input depth. The proposed GPFM follows this objective by suppressing irrelevant RGB textures before depth reconstruction. Unlike flow-based alignment or contrastive refinement, GPFM performs lightweight filtering through geometric prior gating.

Recent studies have further shifted GDSR and RGB-D enhancement from fixed-degradation reconstruction toward degradation-aware, geometry-aware, and prior-guided modeling. DORNet [24] addresses blind depth super-resolution by learning implicit degradation representations and selectively propagating RGB information according to degradation priors. DuCos [25] introduces foundation model prompts and formulates depth super-resolution under a duality-constrained optimization framework, improving robustness across diverse degradation scenarios. GDNet [26] studies compressed depth map super-resolution and decouples the reconstruction process into fine geometric detail learning and global geometric feature extraction. DEPTHOR [27] focuses on practical dToF depth enhancement, showing that real sensor degradation, RGB–depth misalignment, and unreliable measurements remain critical issues for RGB-guided depth reconstruction. Beyond GDSR, DFormerv2 [28] explicitly uses depth maps as geometry priors for RGB-D representation learning, indicating that geometry prior modeling has become an important direction in recent RGB-D research. These recent works suggest that robust RGB-D reconstruction should not rely only on direct feature fusion; instead, degradation characteristics, geometric consistency, and high-frequency structural priors should be explicitly considered.

### 2.3. High-Frequency Structure Modeling

Boundaries, fine details, and local geometric variations in depth maps are typically associated with high-frequency components; therefore, high-frequency recovery is critical for depth map super-resolution. DCTNet [7] reconstructs HR depth features in the discrete cosine domain and separates shared and modality-specific information through semi-coupled feature extraction. This method shows that frequency-domain representations can improve reconstruction and help explain cross-modal texture mis-transfer. Qiao et al. [20] combine explicit and implicit high-frequency features with local–global context and frequency-domain projections for progressive HR depth recovery. RSAG [15] improves high-frequency guidance through adaptive high–low-frequency separation and recurrent structure attention. These studies show that high-frequency information is important for depth structure recovery. However, frequency-domain modeling should go beyond simple compensation and identify high-frequency components that correspond to true geometric structures.

SGNet [21] introduces both gradient-domain and frequency-domain modeling into GDSR. It uses the Gradient Calibration Module (GCM) to calibrate LR depth structures with RGB gradient priors and the Frequency Awareness Module (FAM) to propagate RGB high-frequency components through recurrent Spectrum Differencing Blocks (SDBs). SGNet extends structure recovery beyond the spatial domain by exploiting gradient and frequency cues, identifying missing high-frequency structures as a key limitation of LR depth maps. However, its frequency-domain supervision is mainly based on amplitude and phase differences and does not explicitly distinguish structural contributions across frequency locations. In depth maps, low-frequency errors in smooth regions and high-frequency errors near boundaries affect geometric quality differently. To address this limitation, we propose RCSL, which constrains the real and imaginary components of the prediction and ground truth spectra in the complex spectral domain and uses radial weighting to emphasize boundary-related high-frequency regions.

## 3. Proposed Method

In this section, we present the proposed FGGNet for RGB-guided depth map super-resolution. We first define the task formulation and notation. We then provide an architecture-level overview of FGGNet based on Figure 1, followed by detailed descriptions of MRGConv, GPFM, and RCSL.

### 3.1. Formulation

Given a low-resolution depth map and a spatially aligned high-resolution RGB image, RGB-guided depth map super-resolution aims to recover the corresponding high-resolution depth map. Let the LR depth map, HR RGB guidance image, predicted HR depth map, and HR ground truth depth map be denoted as $D^{lr}\in {\mathbb{R}}^{H\times W\times 1}$, $I^{rgb}\in {\mathbb{R}}^{sH\times sW\times 3}$, $D^{sr}\in {\mathbb{R}}^{sH\times sW\times 1}$, $D^{hr}\in {\mathbb{R}}^{sH\times sW\times 1}$ respectively. Here, *H* and *W* denote the height and width of the LR depth map, and *s* denotes the upsampling factor. The bicubically upsampled LR depth map is denoted as $D_{lr}^{up}\in {\mathbb{R}}^{sH\times sW\times 1}$.

### 3.2. Overall Network Architecture

Figure 1 shows the overall architecture of FGGNet. The network contains an RGB guidance branch, a depth reconstruction branch, three iterative fusion stages, and a reconstruction module. The RGB branch receives $I^{rgb}$ and progressively enhances guidance features through MRGConv. The depth branch receives $D^{lr}$, extracts depth features at the LR resolution, and reconstructs the HR depth map through multi-stage RGB–depth fusion. Instead of extracting all RGB features first and fusing them only once, FGGNet performs iterative cross-modal interaction: after each MRGConv stage, the current RGB feature is filtered and fused with the corresponding depth feature through GPFM, and the updated RGB feature is passed to the next stage.

At the beginning of each fusion stage, the current depth feature is augmented with a gradient-enhanced depth prior. Following SGNet [21], we use the Gradient Calibration Module (GCM) as a gradient prior extractor. SGNet maps the RGB image and the upsampled LR depth map into the gradient domain and uses the RGB gradient prior to calibrate the blurry LR depth gradient. Specifically, the RGB and depth gradient representations are computed as

(1) $$G_{rgb}=f_{gm}(I^{rgb}),\;G_{lr}=f_{gm}(D_{lr}^{up}),$$

where $f_{gm}(\cdot )$ denotes the gradient mapping function, $G_{rgb}$ is the RGB gradient prior, and $G_{lr}$ is the gradient representation of the upsampled LR depth map. The calibrated gradient feature is then obtained by

(2) $$G_{sr}=f_{g}\left(G_{rgb},\;G_{lr}\right),$$

where $f_{g}(\cdot )$ denotes the gradient calibration mapping in GCM. The gradient-enhanced depth prior is defined as

(3) $$G_{d}=f_{ds}\left(f_{r}(D_{lr}^{up})+f_{r}(G_{sr})\right),$$

where $f_{r}(\cdot )$ denotes residual feature extraction and $f_{ds}(\cdot )$ denotes the downsampling projection. This notation follows the role of $F_{ge}$ in SGNet, but we denote the feature as $G_{d}$ to emphasize its use as a depth-derived geometric prior in FGGNet.

Given the enhanced RGB feature and the gradient-augmented depth feature, GPFM first derives a geometric prior from the current depth representation and uses it to gate the RGB feature. This step suppresses RGB texture responses that are inconsistent with the current depth geometry. The filtered RGB feature and the depth feature are then fused in spatial and frequency domains in parallel. After three iterative fusion stages, multi-stage depth features are aggregated and passed to the reconstruction module. The final residual output is added to $D_{lr}^{up}$ to obtain $D^{sr}$.

#### 3.2.1. Multi-Branch RGB-Guided Convolution

The purpose of MRGConv is to enhance RGB structural representations before RGB–depth fusion. Its design is inspired by the multi-branch training topology of RepVGG [29], but the objective is different. RepVGG uses structural re-parameterization to convert training-time multi-branch blocks into a single-branch inference-time convolutional structure. In contrast, MRGConv is used as an RGB guidance-enhancement module in FGGNet and keeps its multi-branch structure during inference. This design is intended to preserve complementary RGB responses rather than to simplify the inference topology.

**Figure 2:**
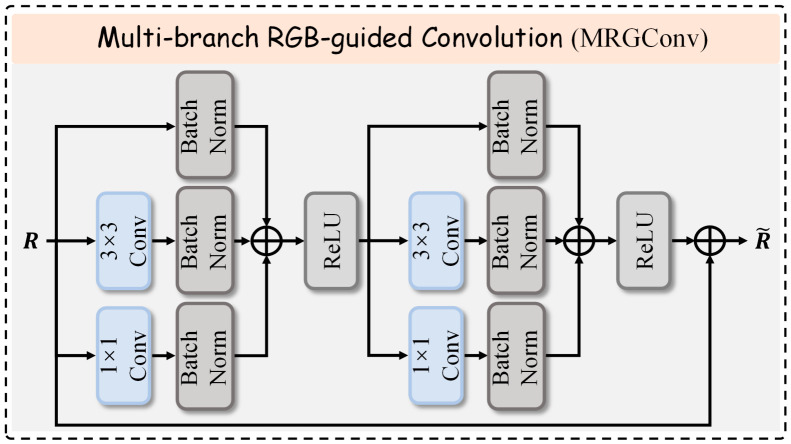
The structure of our proposed Multi-branch RGB-guided Convolution (MRGConv). The circled plus symbol (⊕) denotes element-wise addition.

Let the input RGB feature of MRGConv at stage *t* be denoted as $R^{\left(t\right)}\in {\mathbb{R}}^{C\times h\times w}$, where *C*, *h*, and *w* denote the channel number, height, and width of the feature map, respectively. As shown in Figure 2, MRGConv consists of two cascaded multi-branch convolutional units. Each unit contains a 3 × 3 convolution branch, a 1 × 1 convolution branch, and an identity branch. We denote the three branch mappings as Conv_3_(·), Conv_1_(·), and Id_bn_(·), respectively. A single multi-branch convolutional unit is defined as

(4) $$\mathrm{MRG}(R^{\left(t\right)})=\phi \left({\mathrm{Conv}}_{3}(R^{\left(t\right)})+{\mathrm{Conv}}_{1}(R^{\left(t\right)})+{\mathrm{Id}}_{\mathrm{bn}}(R^{\left(t\right)})\right),$$

where Conv_3_(·) denotes a 3 × 3 convolution followed by batch normalization, and Conv_1_(·) denotes a 1 × 1 convolution followed by batch normalization. Id_bn_(·) denotes an identity branch with batch normalization. It does not perform spatial convolution; instead, it directly normalizes and passes through the input feature. In MRGConv, the input and output channel dimensions are kept identical, so this identity branch can be applied without an additional projection layer. Therefore, this branch preserves the original RGB response and reduces detail loss caused by excessive feature transformation. *ϕ*(·) denotes the ReLU activation function. With two multi-branch convolutional units stacked sequentially, the output of MRGConv is defined as

(5) $${\tilde{R}}^{\left(t\right)}=R^{\left(t\right)}+{\mathrm{MRG}}^{\left(2\right)}({\mathrm{MRG}}^{\left(1\right)}(R^{\left(t\right)})),$$

where MRG^(1)^ and MRG^(2)^ denote the first and second multi-branch convolutional units, respectively, and ${\tilde{R}}^{\left(t\right)}$ denotes the enhanced RGB feature. The outer residual connection preserves the input RGB representation and stabilizes feature refinement.

Unlike RepVGG-style structurally re-parameterized blocks, MRGConv is retained as a multi-branch module at both training and inference stages, because its role in FGGNet is to preserve complementary RGB structural responses rather than to reduce the inference topology to a single convolution.

#### 3.2.2. Geometry Prior-Guided Fusion Module

**Figure 3:**
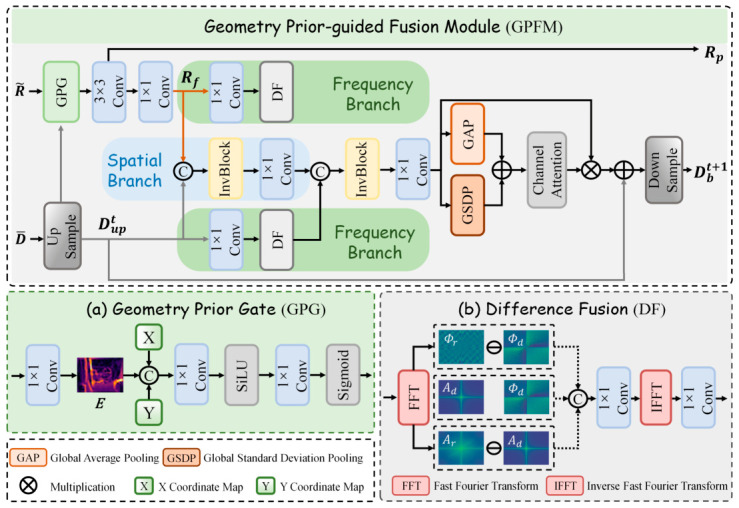
Structure of the proposed Geometry Prior-Guided Fusion Module (GPFM). The circled C symbol (©) denotes channel-wise concatenation.

Enhancing RGB features alone is insufficient to guarantee reliable RGB-guided depth reconstruction. The key issue is not the lack of RGB structural information, but the indiscriminate transfer of RGB high-frequency responses into the depth branch. Such transfer may introduce texture-copying artifacts when RGB edges are inconsistent with true depth discontinuities. To address this problem, GPFM first derives a geometric prior from the current depth representation and uses it to filter the RGB feature before fusion. It then performs coordinated spatial-domain and frequency-domain interactions, as shown in Figure 3.

Let the depth backbone feature at stage *t* be denoted as $D_{b}^{\left(t\right)}\in {\mathbb{R}}^{C\times h\times w}$, where *C*, *h*, and *w* denote the channel number, height, and width of the depth feature, respectively. Before being fed into GPFM, $D_{b}^{\left(t\right)}$ is concatenated with the gradient-enhanced depth prior $G_{d}$ and passed through a channel-alignment mapping:

(6) $${\bar{D}}^{\left(t\right)}=C_{g}\left(\left[D_{b}^{\left(t\right)},G_{d}\right]\right),$$

where [⋅,⋅] denotes channel-wise concatenation, $C_{g}(\cdot )$ denotes the channel-alignment mapping, and ${\bar{D}}^{\left(t\right)}$ denotes the gradient-calibrated depth feature. Compared with $D_{b}^{\left(t\right)}$, ${\bar{D}}^{\left(t\right)}$ contains both the current depth representation and the gradient-enhanced structural prior. Next, ${\bar{D}}^{\left(t\right)}$ is upsampled to the spatial resolution of the enhanced RGB feature ${\tilde{R}}^{\left(t\right)}$:

(7) $$D_{up}^{\left(t\right)}=f_{up}\left({\bar{D}}^{\left(t\right)}\right),$$

where $f_{up}(\cdot )$ denotes the upsampling mapping and $D_{up}^{\left(t\right)}$ denotes the upsampled depth feature. The upsampled depth feature is compressed into a single-channel response map:

(8) $$M^{\left(t\right)}=P_{d}\left(D_{up}^{\left(t\right)}\right),$$

where $P_{d}(\cdot )$ denotes a 1 × 1 channel-compression projection. First-order difference responses are then extracted from $M^{\left(t\right)}$ along the horizontal and vertical directions to construct a normalized geometric edge prior:

(9) $$E^{\left(t\right)}=\frac{\left|\nabla_{x}M^{\left(t\right)}\right|+\left|\nabla_{y}M^{\left(t\right)}\right|}{\max\;\left(\left|\nabla_{x}M^{\left(t\right)}\right|+\left|\nabla_{y}M^{\left(t\right)}\right|\right)+\varepsilon },$$

where $\nabla_{x}(\cdot )$ and $\nabla_{y}(\cdot )$ denote first-order difference operators along the horizontal and vertical directions, respectively. *ɛ* is a small constant used to avoid division by zero. $E^{\left(t\right)}\in [0,1]$ highlights depth structure variations and provides an explicit geometry cue for RGB guidance filtering. The geometric edge map and normalized coordinate maps are then concatenated along the channel dimension to construct the geometric prior:

(10) $$P^{\left(t\right)}=\left[E^{\left(t\right)},X,Y\right],$$

where *X* and *Y* denote the normalized coordinate maps along the horizontal and vertical directions, respectively. *X* and *Y* provide spatial location information, while $E^{\left(t\right)}$ provides depth structure information. Based on $P^{\left(t\right)}$, the RGB gating map is generated by two pointwise mapping layers:

(11) $$A^{\left(t\right)}=\sigma \left(P_{\;2}\left(\mathrm{SiLU}\left(P_{1}(P^{\left(t\right)})\right)\right)\right),$$

where $P_{1}(\cdot )$ and $P_{2}(\cdot )$ denote two 1 × 1 pointwise projections, SiLU(⋅) denotes the SiLU activation function, and σ(⋅) denotes the sigmoid function. The output $A^{\left(t\right)}\in [0,1]$ is the geometry prior gating map. The gating map is applied to the enhanced RGB feature as

(12) $${\hat{R}}^{\left(t\right)}={\tilde{R}}^{\left(t\right)}⊙(1+\alpha (2A^{\left(t\right)}-1)),$$

where ⊙ denotes element-wise multiplication, *α* denotes the gating-intensity coefficient, and ${\hat{R}}^{\left(t\right)}$ denotes the RGB feature after geometric prior filtering. The modulation factor $1+\alpha (2A^{\left(t\right)}-1)$ adaptively enhances or suppresses RGB responses according to the current depth geometry. RGB structures that are consistent with depth discontinuities are retained, whereas texture responses that are inconsistent with the current depth geometry are suppressed before cross-modal fusion. To avoid confusion with the branch operators used in MRGConv, we use $f_{loc}(\cdot )$ and $f_{pre}(\cdot )$ to denote the local reorganization and pre-mapping operations applied to the filtered RGB feature:

(13) $$R_{p}^{\left(t\right)}=f_{loc}({\hat{R}}^{\left(t\right)}),$$

(14) $$R_{f}^{\left(t\right)}=f_{pre}(R_{p}^{\left(t\right)}),$$

where $f_{loc}(\cdot )$ is implemented by a 3 × 3 convolution and $R_{p}^{\left(t\right)}$ is passed to the next RGB branch stage. $f_{pre}(\cdot )$ is implemented by a 1 × 1 convolution and produces $R_{f}^{\left(t\right)}$, which is used for intra-stage RGB–depth fusion.

GPFM employs a dual-path interaction scheme to jointly model local spatial correspondences and high-frequency restoration cues. The spatial path learns local correspondences between depth and RGB features, which are formulated as

(15) $$S^{\left(t\right)}=F_{\mathrm{spa}}^{\left(t\right)}\left(\left[D_{up}^{\left(t\right)},R_{f}^{\left(t\right)}\right]\right),$$

where $F_{\mathrm{spa}}^{\left(t\right)}(\cdot )$ denotes the spatial-domain fusion mapping at stage *t*, and $S^{\left(t\right)}$ denotes the spatially fused feature. Specifically, $F_{\mathrm{spa}}^{\left(t\right)}(\cdot )$ consists of an invertible block and a 1 × 1 compression projection. The invertible block first performs channel mixing on the concatenated depth and RGB features, then splits the mixed feature into two groups and performs cross-group information exchange through coupled transformations. The resulting feature is finally compressed to the target channel dimension by the 1 × 1 projection.

The frequency-domain path complements the spatial-domain path by explicitly modeling high-frequency discrepancies between depth and RGB features. The depth feature and the filtered RGB feature are first mapped into the spectral domain:

(16) $$Q_{d}^{\left(t\right)}=T\left(P_{d}(D_{up}^{\left(t\right)})\right),$$

(17) $$Q_{r}^{\left(t\right)}=T\left(P_{r}(R_{f}^{\left(t\right)})\right),$$

where T(⋅) denotes the two-dimensional fast Fourier transform (FFT), and $P_{d}(\cdot )$ and $P_{r}(\cdot )$ denote the pre-mapping operations for the depth and RGB features before spectral transformation, respectively. The complex spectra are further decomposed into amplitude and phase as follows:

(18) $$Q_{d}^{\left(t\right)}=A_{d}^{\left(t\right)}\exp\left(j\Phi_{d}^{\left(t\right)}\right),$$

(19) $$Q_{r}^{\left(t\right)}=A_{r}^{\left(t\right)}\exp\left(j\Phi_{r}^{\left(t\right)}\right),$$

where $A_{d}^{\left(t\right)}$ and $A_{r}^{\left(t\right)}$ denote the spectral amplitudes of the depth and RGB features, respectively; $\Phi_{d}^{\left(t\right)}$ and $\Phi_{r}^{\left(t\right)}$ denote the corresponding phases; and **j** is the imaginary unit. The amplitude mainly reflects the strength distribution of spectral responses, while the phase preserves structural arrangement and spatial localization information. To preserve the spectral basis of the current depth branch and measure RGB–depth discrepancies in the frequency domain, we perform difference-driven fusion on the amplitude and phase components separately:

(20) $${\bar{A}}^{\left(t\right)}=\Psi_{a}^{\left(t\right)}\left(A_{\;d}^{\;(t)},\left|A_{r}^{\left(t\right)}-A_{d}^{\left(t\right)}\right|\right),$$

(21) $${\bar{\Phi }}^{\left(t\right)}=\Psi_{\phi }^{\left(t\right)}\left(\Phi_{\;d}^{\;(t)},\left|\Phi_{r}^{\left(t\right)}-\Phi_{d}^{\left(t\right)}\right|\right),$$

where $\Psi_{a}^{\left(t\right)}(\cdot )$ and $\Psi_{\phi }^{\left(t\right)}(\cdot )$ denote the amplitude fusion map and phase fusion map at stage *t*, respectively. The absolute difference terms explicitly encode the spectral discrepancy between RGB and depth features. Instead of directly replacing the depth spectrum with the RGB spectrum, this design uses RGB information as a residual spectral cue conditioned on the current depth representation. The fused amplitude and phase are then reconstructed into a complex spectrum and mapped back to the spatial domain:

(22) $$F^{\left(t\right)}=T^{-1}\left({\bar{A}}^{\;(t)}⊙\exp\left(j{\bar{\Phi }}^{\left(t\right)}\right)\right),$$

where $T^{-1}(\cdot )$ denotes the inverse fast Fourier transform (IFFT). The spatially fused feature $S^{\left(t\right)}$ and the frequency-enhanced feature $F^{\left(t\right)}$ are then combined to produce an intermediate spatial–frequency representation:

(23) $$H^{\left(t\right)}=F_{sf}^{\left(t\right)}\left(\left[S^{\left(t\right)},F^{\left(t\right)}\right]\right),$$

where $F_{sf}^{\left(t\right)}(\cdot )$ denotes the spatial–frequency fusion mapping at stage *t*. It is implemented by an invertible block followed by a 1 × 1 compression projection. The invertible block exchanges information between spatial-domain and frequency-domain features, while the compression projection maps the fused representation to the target channel dimension. To select stable channel responses after spatial–frequency fusion, $H^{\left(t\right)}$ is recalibrated along the channel dimension:

(24) $$W^{\left(t\right)}=\sigma \left(\varphi^{\left(t\right)}\left(\mathrm{Std}(H^{\left(t\right)})+\mathrm{Avg}(H^{\left(t\right)})\right)\right),$$

where Std(·) and Avg(·) denote global standard deviation pooling and global average pooling, respectively. $\varphi^{\left(t\right)}(\cdot )$ denotes a channel-wise mapping implemented by two 1 × 1 convolution layers with an intermediate LeakyReLU activation, and $W^{\left(t\right)}$ denotes the channel recalibration weights. The fusion-enhanced depth feature at stage *t* is obtained by residual refinement:

(25) $${\tilde{D}}_{up}^{\left(t\right)}=W^{\left(t\right)}⊙H^{\left(t\right)}+D_{up}^{\left(t\right)},$$

the residual term $D_{up}^{\left(t\right)}$ preserves the structural basis of the current depth branch, while $W^{\left(t\right)}⊙H^{\left(t\right)}$ injects reliable spatial–frequency correction responses. Finally, the output is projected back to the backbone resolution and used as the depth input for the next stage:

(26) $$D_{b}^{\left(t+1\right)}=f_{ds}\left({\tilde{D}}_{up}^{\left(t\right)}\right).$$

Mechanistically, GPFM does not simply concatenate RGB and depth features followed by a convolution. It first derives a geometric prior from the current depth representation to suppress unreliable RGB textures, then models spatial and frequency interactions in parallel, and finally selects reliable fusion responses through channel recalibration. In this way, cross-modal fusion is transformed from indiscriminate RGB information injection into a geometry-constrained and frequency-aware refinement process.

#### 3.2.3. Radial Complex Spectral Loss

Spatial-domain supervision can constrain global numerical errors, but it provides limited guidance for high-frequency structure recovery. In depth map super-resolution, visual quality and geometric accuracy are often determined less by low-frequency errors in smooth regions than by high-frequency recovery near boundaries and local geometric variations. If all frequency locations are treated equally, the optimization tends to be dominated by low-frequency components, which account for a larger proportion of the signal, leaving high-frequency regions insufficiently constrained. Based on this observation, we propose radial complex spectral loss (RCSL), which constrains the discrepancy between the predicted and ground truth depth maps in the complex spectral domain and emphasizes high-frequency regions through radial weighting.

Let ξ=(u,v)∈Ω denote a frequency index, where Ω is the set of half-spectrum frequency indices. In Figure 1, DC(0,0) denotes the Direct Current component, namely the zero-frequency component of the two-dimensional spectrum. The predicted and ground truth spectra are first written as

(27) $$T(D^{sr})(\xi )={Re}^{\prime }(\xi )+jIm^{\prime }(\xi ),$$

(28) T(Dhr)(ξ)=Re(ξ)+jIm(ξ),

where $Re^{\prime }(\xi )$ and Re(ξ) denote the real components of the predicted and ground truth spectra at frequency index *ξ*, respectively; $Im^{\prime }(\xi )$ and Im(ξ) denote the corresponding imaginary components. RCSL is defined as

(29) Lrcsl=1|Ω|∑ξ∈Ωω(ξ)Re′(ξ)−Re(ξ)+Im′(ξ)−Im(ξ),

where |Ω| denotes the number of elements in Ω, and *ω*(*ξ*) denotes the radial weight at frequency index *ξ*, defined as

(30) $$\omega (\xi )=\frac{{\left\|\xi \right\|}_{2}}{\underset{\xi \in \Omega }{\max}{\left\|\xi \right\|}_{2}+\varepsilon },$$

where ${\left\|\xi \right\|}_{2}$ denotes the Euclidean distance from frequency index *ξ* to the spectral origin.

#### 3.2.4. Other Loss Functions

For spatial-domain supervision, we use the mean absolute error (MAE) to constrain pixel-level differences between the predicted and HR ground truth depth maps. To further preserve gradient structures, we introduce a gradient-aware loss. The two losses are defined as

(31) $$L_{spa}=\frac{1}{N}\sum_{i=1}^{N}{\left\|D_{i}^{sr}-D_{i}^{hr}\right\|}_{\;1},$$

(32) $$L_{gra}=\frac{1}{N}\sum_{i=1}^{N}{\left\|G_{i}^{sr}-G_{i}^{hr}\right\|}_{\;1},$$

where *N* denotes the number of elements over which the loss is averaged; $G^{sr}$ and $G^{hr}$ denote the gradient feature and the ground truth gradient, respectively. The total training loss is defined as

(33) $$L_{\mathrm{total}}=L_{spa}+\lambda_{1}L_{gra}+\lambda_{2}L_{rcsl},$$

where *λ*_1_ and *λ*_2_ are hyperparameters that balance the gradient-aware and radial complex spectral losses, respectively.

## 4. Experiments

### 4.1. Datasets

We evaluate FGGNet on four public datasets: NYU v2 [30], Middlebury [31], Lu [32], and RGB-D-D [2]. NYU v2, Middlebury, and Lu are commonly used for synthetic degradation evaluation, whereas RGB-D-D is used for both cross-dataset testing and real-world degradation evaluation. These datasets cover both synthetic and real-world degradation settings and are widely used in guided depth map super-resolution.

In the synthetic setting, LR depth maps are generated by applying bicubic downsampling to the corresponding HR depth maps. FGGNet is trained on the NYU v2 dataset. Following previous studies [7,21], NYU v2 is split into 1000 RGB-D pairs for training and 449 pairs for testing. To evaluate generalization, the model trained on NYU v2 is tested on Middlebury, Lu, and RGB-D-D, containing 30, 6, and 405 test pairs, respectively.

For real-world evaluation, following the protocol in [2], FGGNet is trained and tested on RGB-D-D, which contains 2215 RGB-D pairs for training and 405 pairs for testing under this setting. Compared with synthetic degradation, real-world DSR is more challenging because LR depth maps contain sensor-dependent noise and missing values. In this setting, LR depth maps captured by the low-power time-of-flight (ToF) camera of a Huawei P30 Pro contain noise and depth holes, better reflecting the reconstruction difficulty under real-world degradation.

### 4.2. Evaluation Metrics

Following previous studies [7,21], we use the root-mean-square error (RMSE) as the quantitative evaluation metric. RMSE measures the pixel-level discrepancy between the reconstructed depth map and the HR ground truth depth map, where a lower value indicates better reconstruction quality. RMSE is defined as

(34) $$\mathrm{RMSE}(D^{sr},D^{hr})=\sqrt{\frac{1}{H\times W}\sum_{i=1}^{H}\sum_{j=1}^{W}{\left(D^{sr}(i,j)-D^{hr}(i,j)\right)}^{2}},$$

where *H* and *W* denote the height and width of the evaluated depth map, respectively.

### 4.3. Experimental Settings

All experiments were implemented in PyTorch 2.8.0 with Python 3.11 and conducted on a single NVIDIA A800 GPU. The model was optimized using Adam with an initial learning rate of 10^−4^, a batch size of 2, and 200 training epochs. A MultiStepLR scheduler was stepped at the iteration level, with milestones set to $5\times 10^{4}$, $1\times 10^{5}$, and $1.6\times 10^{5}$ iterations and a decay factor of 0.2. For data preprocessing, training samples were cropped to 256 × 256, and basic data augmentation was applied. The hyperparameters *α*, *λ*_1_, and *λ*_2_ were set to 0.5, 0.001, and 0.002, respectively.

### 4.4. Results

**Figure 4:**
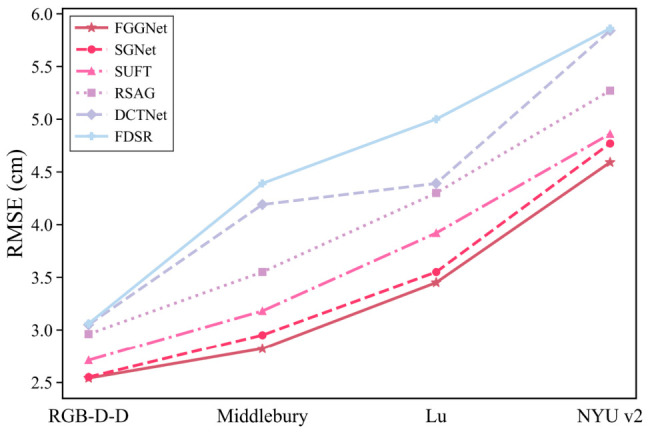
RMSE comparison between FGGNet and existing methods on four benchmarks under the ×16 setting. Lower values indicate better reconstruction accuracy.

We compare FGGNet with representative state-of-the-art methods, including TGV [33], SDF [34], DJF [35], DJFR [36], DMSG [37], PAC [38], DKN [39], FDKN [39], GbFT [40], FDSR [2], JIIF [6], CTKT [5], DCTNet [7], AHMF [4], RSAG [15], SUFT [8], and SGNet [21]. As shown in Figure 4, FGGNet achieves the lowest RMSE among the compared methods on RGB-D-D, Middlebury, Lu, and NYU v2 under the ×16 setting.

#### 4.4.1. Comparison on the NYU v2 Dataset

**Figure 5:**
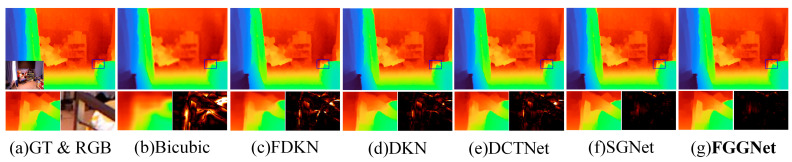
Visual results (left) and error maps (right) on NYU v2 dataset (×8). The blue rectangles indicate the local regions selected for magnified visualization. Brighter colors in the error maps indicate larger errors.

Table 1 reports the quantitative results of FGGNet and existing state-of-the-art DSR methods on the NYU v2 dataset under the ×4, ×8, and ×16 scaling factors, where RMSE is measured in centimeters. As shown in Table 1, FGGNet achieves the lowest RMSE on the NYU v2 dataset under all scaling factors. Compared with the second-best method, FGGNet reduces RMSE by 0.07 cm under the ×8 setting and by 0.18 cm under the more challenging ×16 setting, corresponding to relative improvements of 2.9% and 3.8%, respectively. Figure 5 shows the visual comparison between FGGNet and other DSR methods on the NYU v2 dataset under the ×8 setting. The visual results show that FGGNet better preserves global structures and restores depth boundaries and fine details, especially under the ×8 setting where structural information is severely degraded.

#### 4.4.2. Comparison on the Middlebury and Lu Datasets

**Figure 6:**
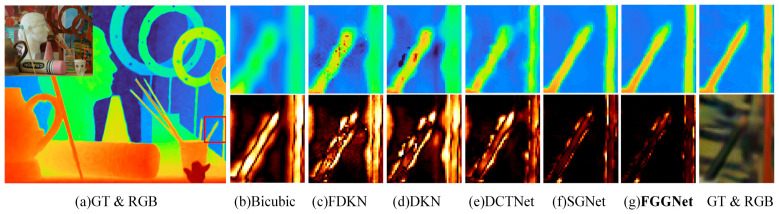
Visual results (top) and error maps (bottom) on Middlebury dataset (×8). The red rectangles indicate the local regions selected for magnified visualization. Brighter colors in the error maps indicate larger errors.

We further evaluate the cross-dataset generalization capability of FGGNet on the Middlebury and Lu datasets. FGGNet is trained on the NYU v2 dataset and directly evaluated on the Middlebury and Lu datasets. Table 2 presents the quantitative comparison results on the Middlebury and Lu datasets. As shown in Table 2, FGGNet achieves competitive results under the ×4 setting and outperforms all compared methods under the ×8 and ×16 settings. Compared with the second-best method under the ×16 setting, FGGNet reduces RMSE by 0.13 cm on Middlebury and 0.10 cm on Lu. Figure 6 displays the visual comparison on the Middlebury dataset under the ×8 setting. The visual results show that FGGNet produces clearer reconstructions in thin structures and edge regions, with lower error responses. These results further demonstrate the effectiveness and cross-dataset generalization capability of FGGNet.

#### 4.4.3. Comparison on the RGB-D-D Dataset

**Figure 7:**
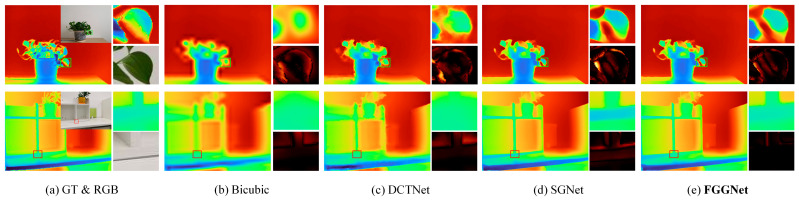
Visual results (top) and error maps (bottom) on RGB-D-D dataset (×16). The red rectangles indicate the local regions selected for magnified visualization. Brighter colors in the error maps indicate larger errors.

To evaluate the effectiveness of FGGNet under real-world degradation, we further conduct experiments on the RGB-D-D dataset. The model trained on the NYU v2 dataset is directly tested on RGB-D-D for cross-dataset evaluation. Table 3 presents the quantitative comparison results on the RGB-D-D dataset. As shown in Table 3, FGGNet achieves the best or second-best RMSE across different super-resolution scales. On the RGB-D-D dataset, FGGNet achieves the best RMSE under the ×4 and ×16 settings, but it is slightly inferior to SGNet under the ×8 setting by 0.02 cm. This result indicates that FGGNet does not comprehensively outperform SGNet at every scale. A possible reason is that the geometry prior gate may suppress part of the RGB high-frequency responses that remain useful under moderate degradation. When the upsampling factor increases to ×16, missing depth structures and texture over-transfer become more severe; under this more challenging setting, geometry-constrained RGB filtering and radial spectral supervision become more beneficial. Therefore, the advantage of FGGNet is more evident under large-scale upsampling and real-world degradation, where reliable guidance selection is more important than direct RGB feature propagation. Figure 7 displays the visual comparison on the RGB-D-D dataset under the ×16 setting. The visual results show that FGGNet reconstructs clearer leaf and bookshelf edges than the compared methods and produces lower responses in the error maps.

**Figure 8:**
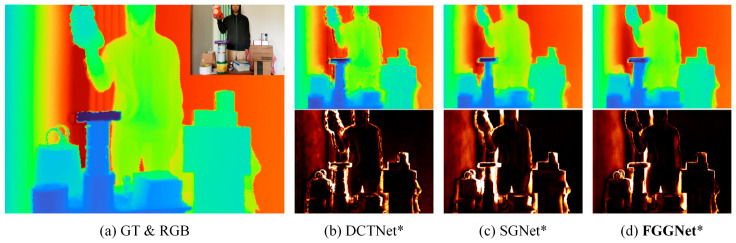
Visual results (top) and error maps (bottom) on the real-world RGB-D-D dataset (×4). * denotes the results obtained after fine-tuning on the real-world RGB-D-D training data. Brighter colors in the error maps indicate larger errors.

To further evaluate the effectiveness of FGGNet in real-world scenarios, we conducted additional experiments on the RGB-D-D dataset. Following the protocol in [2], we first evaluated the pretrained ×4 model from Table 3 on RGB-D-D without fine-tuning, and then retrained and tested the model on the RGB-D-D dataset. As shown in Table 4 and Figure 8, FGGNet achieves the best performance both before and after retraining, demonstrating its robustness under real-world degradation.

#### 4.4.4. Additional Evaluation and Statistical Significance Analysis

To provide a more comprehensive evaluation, we further report mean absolute error (MAE) and Structural Similarity Index Measure (SSIM) for DCTNet, SGNet, and FGGNet on NYU v2, Middlebury, and RGB-D-D. MAE measures the average absolute depth deviation between the reconstructed depth map and the ground truth depth map, while SSIM evaluates structural consistency. MAE is a lower-is-better metric, whereas SSIM is a higher-is-better metric. All methods are evaluated using the same testing pipeline, including identical test images, cropping strategy, depth-value recovery, and metric computation. Since RMSE has already been reported in the main quantitative comparison tables, Table 5 focuses on the supplementary MAE and SSIM results.

In addition, paired Wilcoxon signed-rank tests are conducted on per-image MAE and SSIM values between FGGNet and each baseline to assess statistical reliability. Specifically, $p_{MAE}^{D}$ and $p_{SSIM}^{D}$ denote the (*p*)-values from the Wilcoxon signed-rank test between FGGNet and DCTNet based on per-image MAE and SSIM, respectively. Similarly, $p_{MAE}^{S}$ and $p_{SSIM}^{S}$ denote the corresponding (*p*)-values between FGGNet and SGNet. A result is considered statistically significant when *p* < 0.05.

As shown in Table 5, FGGNet achieves lower MAE and higher SSIM than DCTNet across all tested datasets and scale factors, and the corresponding paired tests show statistically significant differences in all cases. Compared with SGNet, FGGNet achieves better or comparable MAE and SSIM results. Significant improvements are observed on NYU v2 under the ×8 and ×16 settings, on Middlebury under most settings, and on RGB-D-D under the ×4 setting. On NYU v2 ×4 and RGB-D-D under the ×8 and ×16 settings, the improvements over SGNet are not statistically significant, indicating that FGGNet and SGNet perform comparably in these cases. These results complement the RMSE comparisons and show that the proposed method improves not only pixel-wise depth accuracy but also structural consistency in most benchmark settings.

### 4.5. Ablation Study

In this section, we conduct ablation experiments on the NYU v2 and Middlebury datasets to evaluate the effectiveness of the proposed components.

The baseline removes the geometry prior gate (GPG) from GPFM in Figure 3, replaces MRGConv with a residual group, and removes RCSL from the total training loss. Our architecture keeps MRGConv and GPFM but removes RCSL, denoted “w/o RCSL”. Our architecture keeps RCSL and GPFM but replaces MRGConv with a residual group, denoted “w/o MRGConv”. Our architecture keeps RCSL and MRGConv but removes the geometry prior gate module, denoted “w/o GPFM”. Our architecture keeps RCSL but removes both MRGConv and GPFM, denoted “w/o MRGConv & GPFM”.

**Figure 9:**
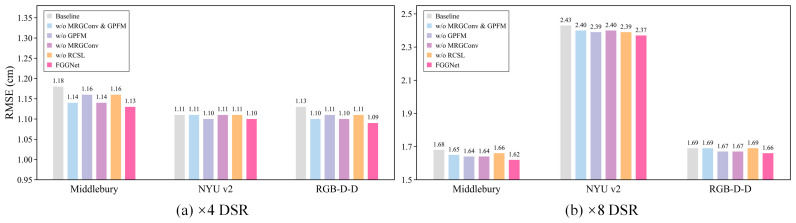
Ablation results of FGGNet on the NYU v2, RGB-D-D and Middlebury datasets. (**a**) Average RMSE for ×4 DSR. (**b**) Average RMSE for ×8 DSR.

As shown in Figure 9, the complete FGGNet generally achieves lower RMSE than the baseline across different datasets and scale factors. Under the ×4 setting, FGGNet reduces the RMSE from 1.18 cm to 1.13 cm on Middlebury, and from 1.13 cm to 1.09 cm on RGB-D-D. The improvement on NYU v2 is smaller, where the RMSE decreases from 1.11 cm to 1.10 cm. This indicates that when the degradation is relatively mild, the benefit of the proposed components is more visible on datasets with sharper structures or real-world degradation than on NYU v2. Under the ×8 setting, FGGNet improves the baseline from 2.43 cm to 2.37 cm on NYU v2, from 1.68 cm to 1.62 cm on Middlebury, and from 1.69 cm to 1.66 cm on RGB-D-D. These results show that the proposed components remain effective under moderate upsampling.

**Figure 10:**
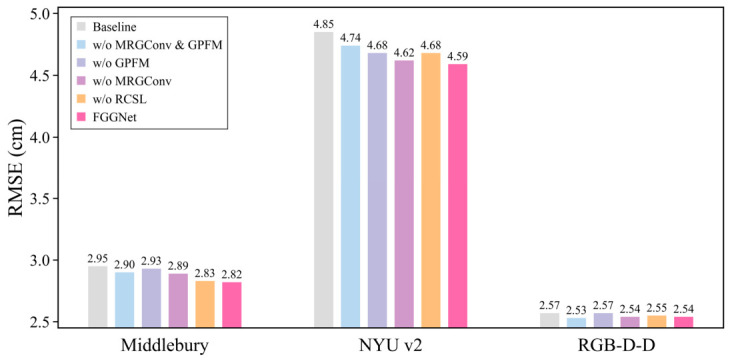
Ablation results of FGGNet on the NYU v2, RGB-D-D and Middlebury datasets (×16).

As shown in Figure 10, the advantage of FGGNet becomes more evident under the ×16 setting. Compared with the baseline, FGGNet reduces the RMSE from 4.85 cm to 4.59 cm on NYU v2, from 2.95 cm to 2.82 cm on Middlebury, and from 2.57 cm to 2.54 cm on RGB-D-D. These results indicate that the proposed frequency–geometry-guided design is more beneficial when the depth degradation becomes severe.

The component-wise comparisons further verify the effectiveness of the proposed design. First, removing RCSL consistently weakens the reconstruction performance in most settings. For example, the RMSE increases from 1.13 cm to 1.16 cm on Middlebury ×4, from 1.62 cm to 1.66 cm on Middlebury ×8, from 4.59 cm to 4.68 cm on NYU v2 ×16. These results indicate that RCSL provides useful frequency-domain supervision, especially for boundary-related high-frequency recovery.

Second, MRGConv and GPFM contribute to the RGB guidance process from different aspects. Removing MRGConv increases the RMSE from 2.37 cm to 2.40 cm on NYU v2 ×8, from 2.82 cm to 2.89 cm on Middlebury ×16, showing that RGB structural enhancement before fusion is beneficial. Removing GPFM leads to larger degradation in several challenging cases, such as Middlebury ×16, where the RMSE increases from 2.82 cm to 2.93 cm, and NYU v2 ×16, where the RMSE increases from 4.59 cm to 4.68 cm. This confirms that geometry prior filtering helps suppress unreliable RGB texture transfer when depth structures are severely degraded.

Finally, the “w/o MRGConv & GPFM” evaluates the joint effect of the two structure-related modules. Compared with FGGNet, removing both modules increases the RMSE from 4.59 cm to 4.74 cm on NYU v2 ×16, and from 1.62 cm to 1.65 cm on Middlebury ×8. These results show that RGB structural enhancement and geometry prior filtering are complementary in most cases. Nevertheless, this variant obtains slightly lower RMSE than FGGNet on RGB-D-D ×16, indicating that geometry-based suppression may occasionally remove useful RGB responses under specific degradation conditions. Therefore, FGGNet is not claimed to be optimal for every individual dataset-scale case; rather, Figure 9 shows that the complete model provides more stable behavior across synthetic and real-world degradation settings.

Table 6 compares the computational efficiency of different module configurations. As shown in Table 6, the performance gain brought by RCSL mainly comes from optimizing the training objective rather than adding inference-time structures. GPFM and MRGConv introduce only lightweight overheads, increasing FLOPs by 1.659 G and 3.391 G and parameters by 0.006 M and 0.010 M, respectively. These increases are marginal relative to the overall computational scale of the baseline. The complete model increases FLOPs and parameters by only approximately 0.17% and 0.04%, respectively, while achieving the best ablation performance. This result indicates that FGGNet improves performance mainly through more effective training constraints, cross-modal structural representation, and geometric prior guidance rather than by substantially increasing model complexity.

**Figure 11:**
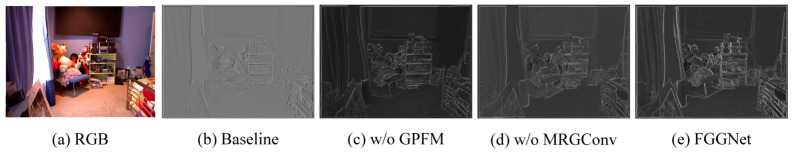
Visualization comparison of the RGB features on NYU v2 dataset (×16). They are derived from the first stage of feature fusion.

In addition, Figure 11 visualizes the RGB feature maps before the first feature fusion stage. Compared with the baseline, MRGConv strengthens structural responses in the feature maps and preserves more high-frequency structural cues. GPFM produces a different response pattern: major structural boundaries are retained while irrelevant texture responses are suppressed, leading to more geometrically consistent boundary features. When MRGConv and GPFM are jointly applied, the resulting feature maps show clearer and more continuous structural responses. These results suggest that FGGNet enables RGB features to provide more reliable structural guidance for GDSR.

## 5. Discussion

SGNet [21] exploits RGB-derived gradient and frequency information for depth structure enhancement. FGGNet follows this gradient–frequency-aware direction but addresses a different issue. SGNet mainly focuses on how to introduce useful RGB structural information into the depth branch, whereas FGGNet further considers whether the introduced RGB responses are geometrically reliable. In RGB-guided depth reconstruction, RGB edges and depth discontinuities are not always aligned. Directly propagating RGB high-frequency responses may therefore cause texture-copying artifacts. For this reason, FGGNet uses GCM only as a gradient prior extractor and introduces GPFM to filter RGB features according to the current depth geometry before fusion. This difference changes the role of RGB guidance. In SGNet, RGB information mainly serves as a structural source for enhancing depth reconstruction. In FGGNet, RGB information is treated as conditionally reliable guidance.

The main strength of FGGNet is that it explicitly models the reliability of RGB guidance. MRGConv improves the structural representation of RGB features, GPFM uses depth-derived geometry to filter RGB responses before fusion, and RCSL strengthens high-frequency supervision around depth boundaries. The revised ablation results in Figure 9 show that these components are generally complementary.

## 6. Conclusions

In this study, we proposed FGGNet, a unified framework for RGB-guided depth map super-resolution, to address insufficient high-frequency structure recovery and RGB texture over-transfer. RCSL was introduced to impose radially weighted constraints in the complex spectral domain, encouraging the recovery of boundary-related high-frequency structures. MRGConv was designed to enhance RGB-guided structural representations before cross-modal fusion. GPFM further used depth-derived geometric priors to filter unreliable RGB guidance and improve the geometric consistency of cross-modal fusion. Experiments on four benchmark datasets showed that FGGNet achieved competitive or superior reconstruction accuracy compared with existing methods. Despite its effectiveness under synthetic and real-world degradation settings, FGGNet still has limitations. First, the geometry prior in GPFM is derived from local depth variations and coordinate maps. When the LR depth input contains severe missing regions, strong sensor noise, or inaccurate local gradients, the generated prior may be less reliable. Second, geometry prior filtering may occasionally suppress useful RGB details, which explains why FGGNet is not always the best in every individual dataset-scale case. Future work will investigate more adaptive geometric prior modeling, sensor-aware degradation simulation, and robustness across different devices and acquisition conditions.

## Figures and Tables

**Table 1:** Quantitative comparison of methods on the NYU v2 dataset. The best result is in bold and the second best is underlined.

Methods	NYU v2		
	×4	×8	×16
Bicubic	8.16	14.22	22.32
TGV [33]	4.98	11.23	28.13
DJF [35]	3.54	6.20	10.21
PAC [38]	3.02	2.99	9.17
GbFT [40]	3.35	5.73	9.01
FDKN [39]	1.86	3.58	6.96
DKN [39]	1.62	3.26	6.51
FDSR [2]	1.61	3.18	5.86
CTKT [5]	1.49	2.73	5.11
DCTNet [7]	1.59	3.16	5.84
AHMF [4]	1.40	2.89	5.64
RSAG [15]	1.23	2.51	5.27
SUFT [8]	1.12	2.51	4.86
SGNet [21]	**1.10**	2.44	4.77
FGGNet (Proposed)	**1.10**	**2.37**	**4.59**

**Table 2:** Quantitative comparison of methods on the Middlebury and Lu datasets. The best result is in bold and the second-best is underlined.

Methods	Middlebury			Lu		
	×4	×8	×16	×4	×8	×16
Bicubic	2.28	3.98	6.37	2.42	4.54	7.38
DJF [35]	1.68	3.24	5.62	1.65	3.96	6.75
DJFR [36]	1.32	3.19	5.57	1.15	3.57	6.77
FDKN [39]	1.08	2.17	4.50	0.82	2.10	5.05
DKN [39]	1.23	2.12	4.24	0.96	2.16	5.11
FDSR [2]	1.13	2.08	4.39	1.29	2.19	5.00
DCTNet [7]	1.10	2.05	4.19	0.88	1.85	4.39
RSAG [15]	1.13	1.74	3.55	**0.79**	1.67	4.30
SUFT [8]	**1.07**	1.75	3.18	1.10	1.74	3.92
SGNet [21]	1.15	1.64	2.95	1.03	1.61	3.55
FGGNet (Proposed)	1.13	**1.62**	**2.82**	1.01	**1.60**	**3.45**

**Table 3:** Quantitative comparison of methods on the RGB-D-D dataset. The best result is in bold and the second best is underlined.

Methods	RGB-D-D		
	×4	×8	×16
Bicubic	2.00	3.23	5.16
SDF [34]	4.06	5.51	7.39
DJF [35]	3.41	5.57	8.15
PAC [38]	1.25	1.98	3.49
DJFR [36]	3.35	5.57	7.99
DKN [39]	1.30	1.96	3.42
FDKN [39]	1.18	1.91	3.41
FDSR [2]	1.16	1.82	3.06
JIIF [6]	1.17	1.79	2.87
DCTNet [7]	**1.08**	1.74	3.05
RSAG [15]	1.14	1.75	2.96
SUFT [8]	1.10	1.69	2.71
SGNet [21]	1.10	**1.64**	2.55
FGGNet (Proposed)	1.09	1.66	**2.54**

**Table 4:** Quantitative comparison of methods on the real-world branch of the RGB-D-D dataset. The best result is in bold and the second best is underlined. * denotes the results obtained after fine-tuning on the real-world RGB-D-D training data.

Methods	Train	RMSE
DJFR [36]	NYU-v2	8.01
DKN [39]	NYU-v2	7.38
FDSR [2]	NYU-v2	7.50
DCTNet [7]	NYU-v2	7.37
SUFT [8]	NYU-v2	**7.22**
SGNet [21]	NYU-v2	**7.22**
FGGNet (Proposed)	NYU-v2	**7.22**
FDSR *	RGB-D-D	5.49
DCTNet *	RGB-D-D	5.41
SUFT *	RGB-D-D	5.41
SGNet *	RGB-D-D	5.32
FGGNet * (Proposed)	RGB-D-D	**5.13**

**Table 5:** Quantitative comparison and paired statistical significance analysis on the NYU v2, Middlebury, and RGB-D-D datasets. ↑ indicates that higher values are better, and ↓ indicates that lower values are better. The best result is in bold.

Dataset	Scale	DCTNet [7]		SGNet [21]		FGGNet		$p_{MAE}^{D}$	$p_{SSIM}^{D}$	$p_{MAE}^{S}$	$p_{SSIM}^{S}$
		MAE ↓	SSIM ↑	MAE ↓	SSIM ↑	MAE ↓	SSIM ↑				
NYU v2	×4	0.79	0.9949	**0.37**	**0.9979**	0.38	**0.9979**	<0.001	<0.001	1	1
	×8	1.82	0.9798	0.94	0.9912	**0.93**	**0.9914**	<0.001	<0.001	<0.001	<0.001
	×16	3.90	0.9572	1.96	0.9786	**1.90**	**0.9796**	<0.001	<0.001	<0.001	<0.001
Middlebury	×4	0.72	0.9727	0.70	0.9699	**0.69**	**0.9707**	0.006	1	<0.001	<0.001
	×8	1.25	0.9457	0.90	0.9627	**0.89**	**0.9634**	<0.001	<0.001	0.035	<0.001
	×16	2.55	0.8985	1.40	0.9438	**1.38**	**0.9447**	<0.001	<0.001	0.006	0.042
RGB-D-D	×4	0.37	0.9889	0.34	0.9892	**0.33**	**0.9897**	<0.001	<0.001	<0.001	<0.001
	×8	0.73	0.9689	**0.53**	0.9773	**0.53**	**0.9774**	<0.001	<0.001	1	0.69
	×16	1.55	0.9366	**0.93**	0.9587	**0.93**	**0.9588**	<0.001	<0.001	0.943	0.858

**Table 6:** Comparison of computational efficiency on NYU v2 dataset (×8).

Methods	Baseline	w/o MRGConv and GPFM	w/o MRGConv	w/o GPFM	All
**Time (ms)**	220	220(+0)	223(+3)	228(+8)	231(+11)
**FLOPs (G)**	3016.095	3016.095(+0)	3017.754(+1.659)	3019.486(+3.391)	3021.145(+5.05)
**Params (M)**	39.925	39.925(+0)	39.931(+0.006)	39.935(+0.010)	39.941(+0.016)

## Data Availability

Publicly available datasets were analyzed in this study. The NYU Depth V2 dataset is available at https://cs.nyu.edu/~silberman/datasets/nyu_depth_v2.html (accessed on 1 December 2025). The Middlebury Stereo datasets are available at https://vision.middlebury.edu/stereo/data/ (accessed on 1 December 2025). The Lu dataset used for depth enhancement evaluation is available at https://web.cecs.pdx.edu/~fliu/project/depth-enhance/ (accessed on 1 December 2025). The RGB-D-D dataset is available at https://github.com/lingzhi96/RGB-D-D-Dataset (accessed on 1 December 2025). No new datasets were created in this study.
